# High Expression of RhoBTB3 Predicts Favorable Chemothrapy Outcomes in non-M3 Acute Myeloid Leukemia

**DOI:** 10.7150/jca.50472

**Published:** 2021-05-17

**Authors:** Shuang-Hui Yang, Wei Liu, Jie Peng, Ya-Jing Xu, Yan-Feng Liu, Yan Li, Min-Yuan Peng, Zhao Ou-Yang, Cong Chen, En-Yi Liu

**Affiliations:** Department of Hematology, XiangYa Hospital, Central South University, XiangYa Road No.87, Changsha 410008, China.

**Keywords:** Rho family GTPases, RhoBTB3, acute myeloid leukemia, prognostic value, TCGA

## Abstract

**Background:** The expression patterns and prognostic significance of the Rho family GTPases in acute myeloid leukemia have not been systematically studied yet.

**Methods:** In our study, we analyzed the expression patterns of 21 Rho family GTPases gene members in AML patients based on GEPIA database. 10 gene members with significant differential expression in AML tissue and healthy tissue were selected for subsequent research. Survival curve analysis in TCGA and GEO dataset preliminary showed that RhoBTB3 is related with the prognosis of non-M3 AML patients. The differential expression of RhoBTB3 on AML bone marrow and normal bone marrow was verified by RT-qPCR. We performed Kaplan-Meier survival analysis and Multivariate Cox analysis to assess the prognostic value of RhoBTB3 in non-M3 AML patients with different treatment regimens. Gene functional enrichment analysis of RhoBTB3 was performed using GO, KEGG and PPI network.

**Results:** The AML patients from TCGA database were partitioned into 2 groups based on different treatment regimens: chemotherapy group and allo-HSCT group. In chemotherapy group, patients with higher expression level of RhoBTB3 showed relatively longer OS and EFS, multivariate Cox analysis revealed high RhoBTB3 mRNA expression as an independent favorable prognostic factor. However, in allo-HSCT group, no significant difference of OS and EFS were found between RhoBTB3 high and low subgroups. Meanwhile, allo-HSCT could circumvent the unfavorable prognosis that was associated with downregulation of RhoBTB3. Functional enrichment analysis showed the association of RhoBTB3 expression with several fundamental physiological components and pathways, including extracellular matrix components, extracellular structure organization, and cytokine-cytokine receptor interaction.

**Conclusions:** Our study identified RhoBTB3 as a prognostic marker and may aid in the selection of the appropriate treatment options between chemotherapy and allo-HCST in non-M3 AML patients. Further researches are necessary to clarify the involvement of RhoBTB3 in the pathogenesis of AML.

## Introduction

Acute myeloid leukemia (AML) is among the most common haematopoietic malignancies. Nowadays, fusion gene expression, gene mutation status, karyotype, and molecular classification may provide information on prognosis and therapeutic outcomes 1. However, there are still many patients who cannot be classified by common indicators. We need to explore more indicators to further distinguish patients with good and poor prognosis and provide personalized treatment. The AML patients typically undergo chemotherapy, demethylation therapy, and transplantation therapy. The application of various new drugs tries to improve the prognosis of patients 2. At the same time, people are also looking for new therapeutic targets.

The Rho family GTPases is found in nearly all eukaryotes. They are part of the Ras superfamily which is divided into two types, typical and atypical, on the basis of their regulation mode. The Rho family GTPases includes 21 members which are grouped into eight subfamilies. RhoA, RhoB, and RhoC are assigned to the Rho subfamily. Rac1, Rac2, Rac3, and RhoG are assigned to the Rac subfamily. Cdc42, RhoJ, and RhoQ are members of the Cdc42 subfamily. RhoD and RhoF are members of the RhoD/RhoF subfamily. Rnd1, Rnd2, and Rnd3 are members of the Rnd subfamily. RhoH is the only member of the RhoH subfamily. RhoU and RhoV are members of the RhoU/RhoV subfamily. RhoBTB1, RhoBTB2, and RhoBTB3 are members of the RhoBTB subfamily. They participate in various kinds of cellular processes, including proliferation, cell cycle regulation, cytoskeletal regulation, polarity as well as migration, and gene expression regulation 3-5. The family genes participate in the pathological process of many diseases 6-9. Furthermore, the Rho family GTPases has attracted increasing interest in research on its potential role in cancer development. For instance, RhoA is highly expressed in multiple myeloma and plays an critica role in chemotaxis and adhesion 10. RhoA frequently mutated in EB-virus positive diffuse large B-cell lymphoma 11. Research shows that in renal cell carcinoma, RhoB may serve a tumor suppressor gene. Overexpression of RhoB inhibited tumor cell proliferation and also facilitated their apoptosis 12. RhoC is associated with angiogenesis and matrix remodeling of liver cancer cells 13. In lung cancer, RhoC participated in the course of epithelial-mesenchymal transition induced by TGF-β1 in cancer cells 14. In breast cancer, overexpression of Rac1 is related to multidrug resistance 15. Previous studies have unveiled that the Rho family GTPases could be a potential target in cancer treatment. However, the Rho family GTPases have not been systematically studied in AML. Therefore, in our study, we determined the expression of Rho family GTPases members in some public databases to determine their clinical value and potential therapeutic value in AML.

## Materials and Methods

### Database Analysis

GEPIA (gepia.cancer-pku.cn) is a database for gene expression profiling and interactive analyses in cancer tissue and healthy tissue 16. The differential expression of Rho family GTPases gene members in AML bone marrow samples (from TCGA data) and healthy donor bone marrow samples (from GTEx data) was compared in GEPIA. TCGA (cancergenome.nih.gov) is a database that contains sequencing data from patients of 33 different tumor types 17. We used TCGAbiolinks to download AML mRNA data 18. Since compared with other subtypes of AML, the M3 subtype AML has its own biological characteristics and favorable outcomes, this study only included non-M3 AML patients. The cBioPortal (www.cbioportal.org) is a website that integrates data from TCGA, CCLE, and several independent large-scale tumor research projects 19. The gene expression and clinical information of non-M3 AML were used for further analysis of the Rho GTPases genes. The GEO database (www.ncbi.nlm.nih.gov/geo) is a database funded and maintained by the National Center for Biotechnology Information. The gene expression profile dataset from GSE71014 was based on an Illumina chip and included 104 samples of AML patients.

### DEGs and PPI analysis

To identify the genes related with RhoBTB3, we further analyzed differentially expressed genes (DEGs) between the RhoBTB3 high and low group according to the median expression level among non-M3 AML cases in TCGA. The edgeR package analyzed DEGs associated with RhoBTB3. For DEGs significance, *P*< 0.05 and fold change>2 (| log2FC |> 1) were recognized as the cut-off criteria.

The volcano plot was drawn with the ggplot2 package. The heat map was drawn with the pheatmap package. The ClusterProfiler R package was for identifying GO and KEGG enrichment 20. We also applied STRING to develop the PPI network 21. The protein interaction was visualized using cytoscape software. *P* < 0.05 was considered to be of significance.

### Statistical analysis

The association between gene expression and clinicopathological characters was tested using the Mann-Whitney test, Fisher's exact test or χ^2^ test. The univariate Cox regression analyses were used for evaluating associations of the variables with OS and EFS. The variables included RhoBTB3 expression level, age, gender, WBC count, and RUNX1, TP53, ASXL1, NPM1, FLT3-ITD, biCEBPA mutation statuses. The multivariate Cox regression analyses were performed to analyse independent prognostic factors for patient survival. In multivariate Cox regression model, above variables were assessed by stepwise analysis (backward:LR). The data was analysed using SPSS 25.0 software (IBM, NY, USA). GraphPad Prism 8 software (GraphPad, San Diego, USA) was used to generate and analyze survival curves (log-rank test). *P*<0.05 was considered to be of significance throughout.

### Bone marrow samples and Quantitative real-time PCR

For further verification, bone marrow samples from 16 diagnosed non-M3 AML patients were collected, and 16 bone marrow samples from nonhematological malignancies conditions such as healthy donors for hematopoietic stem cell transplantation, iron deficiency anemia, megaloblastic anemia, or idiopathic thrombocytopenic purpura were collected as comparison. This study was authorized by Medical Ethics Committee of Xiangya hospital, Central South University. All patients signed informed consents. Total RNA of samples was isolated using TRIzol reagent (Invitrogen) and then reversely transcribed using reverse transcription kit (TaKaRa). Primers for real-time PCR were from Sangon Biotech. The sequences of primer were as follows: RhoBTB3 forward 5'-CCGAGATGTACCAAGTGTCCAG-3', RhoBTB3 reverse 5'-GCCAGGTTGAAAGGCAATCAGAG-3', Actin forward 5'-CCATCATGAAGTGTGACG-3', Actin reverse 5'-GCCGATCCACACGGAGTA-3'.

## Results

### Rho family GTPases genes are differentially expressed in AML bone marrow and normal bone marrow

**Figure 1:**
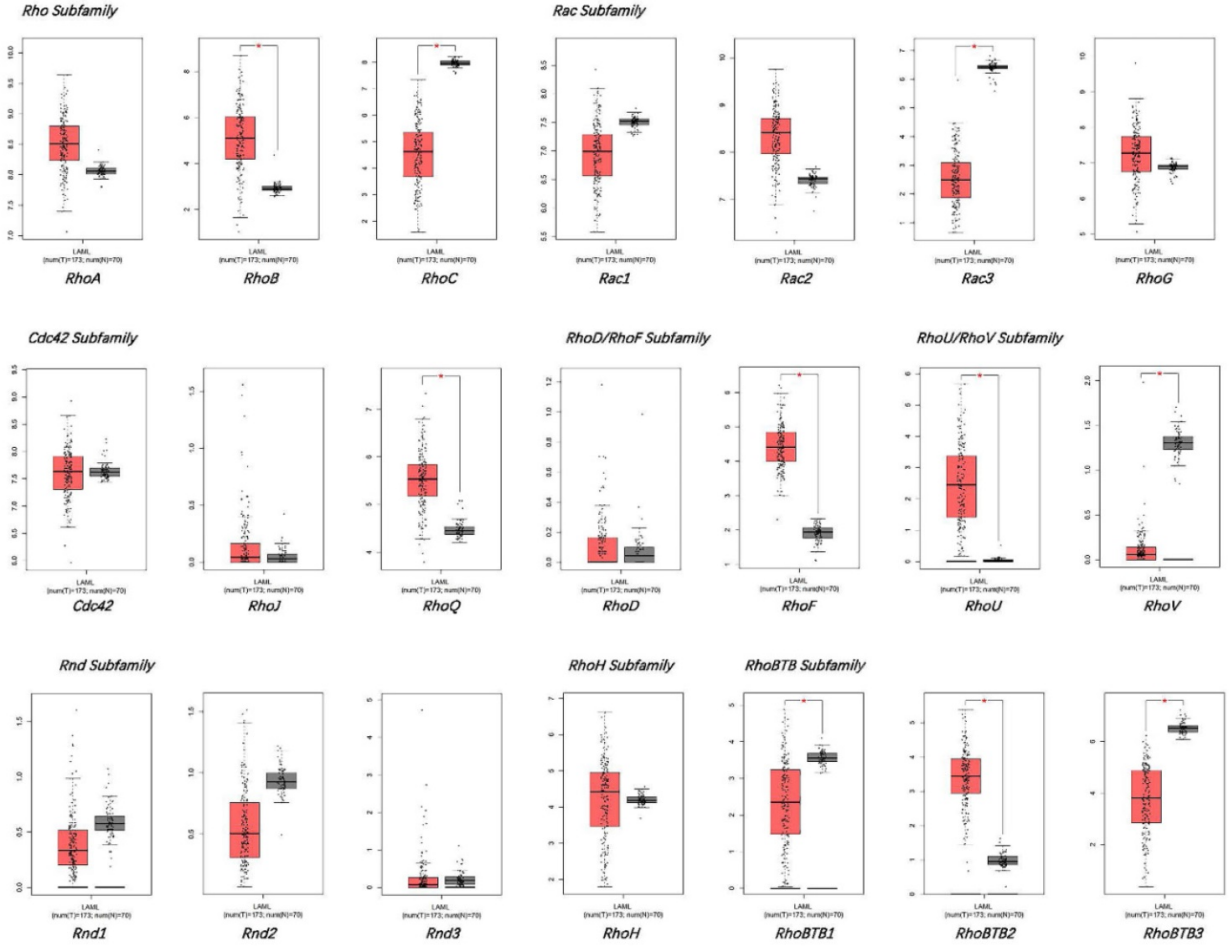
The expression of Rho family GTPases gene members in AML and healthy controls.

GEPIA database was used for comparing the mRNA levels of the Rho family GTPases genes between bone marrow samples of AML patients and healthy donors (**Figure 1**). The results displayed that the mRNA expression levels of Rac3, RhoBTB1, RhoBTB3, RhoC, and RhoV significantly decreased in AML bone marrow, while the mRNA expression levels of RhoB, RhoBTB2, RhoF, RhoQ, and RhoU significantly increased in AML bone marrow compared to healthy samples (*P* < 0.05). The expression level of other genes in the Rho family GTPases was not significantly different between the AML bone marrow tissues and the corresponding healthy tissues in the GEPIA database. These findings suggest that the significant differential expressed genes may be associated with AML tumorigenesis and progression.

### RhoBTB3 may be a potential biomarker for AML prognosis

**Figure 2:**
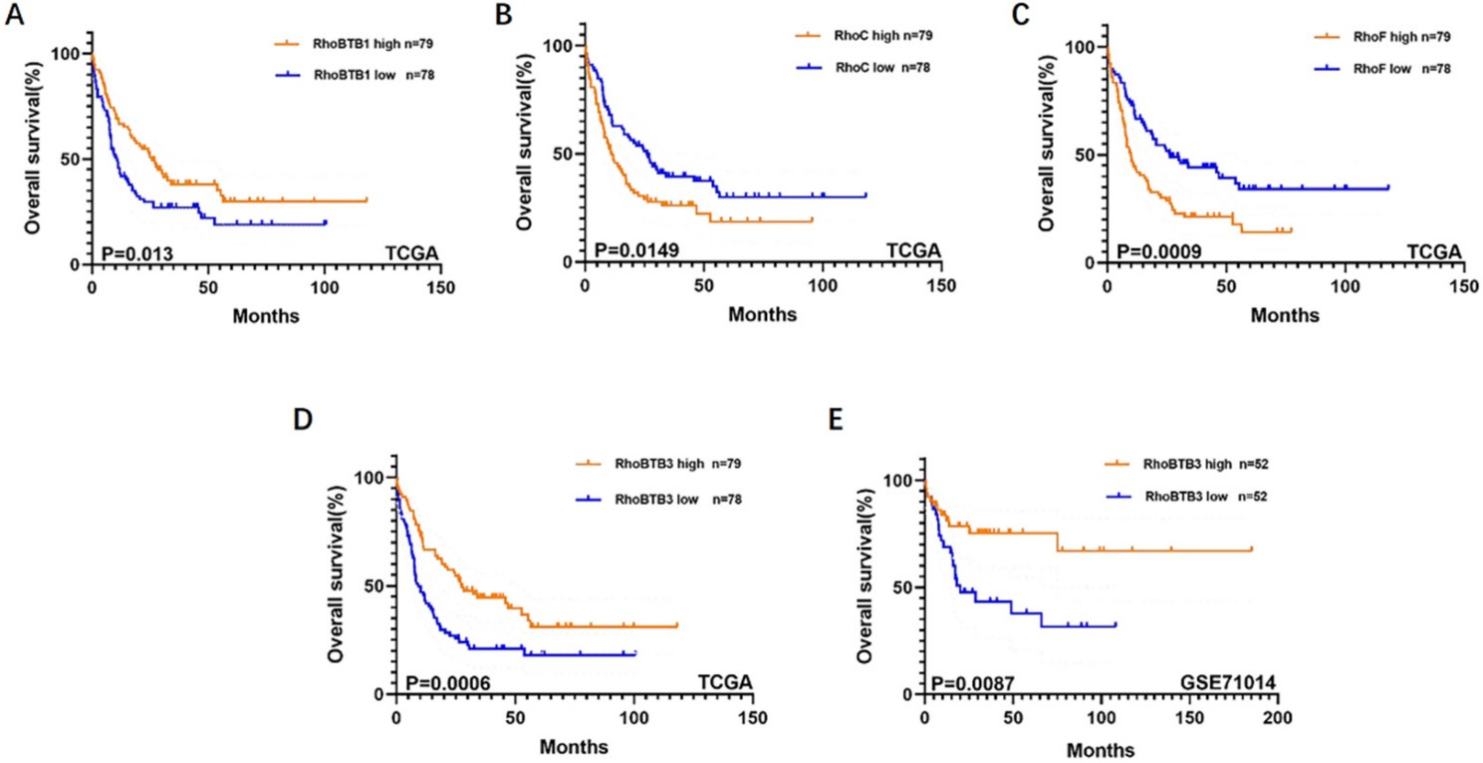
** Kaplan-Meier survival analysis on relationship between different genes and OS of non-M3 AML patients. RhoBTB1 (A)**, RhoC **(B)**, RhoF **(C)**, and RhoBTB3 **(D)** expression levels were significantly associated with OS in the TCGA. RhoBTB3 **(E)** expression levels was significantly associated with OS in the GSE71014.

**Figure 3:**
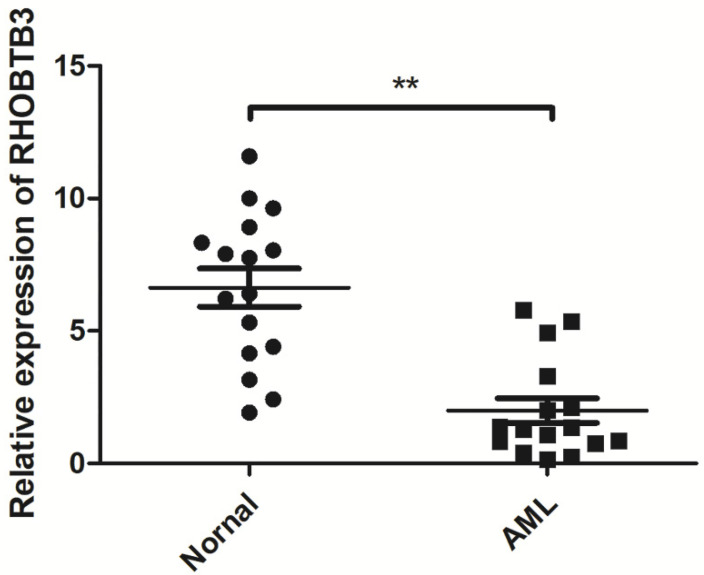
The expression of RhoBTB3 mRNA in AML and controls.

To further explore the role of the Rho family GTPases gene members in the survival of non-M3 AML patients, we selected the aforementioned Rho family GTPases genes with significant differential expression in AML tissue and healthy tissue for subsequent research. Using TCGA data, we performed survival curve analysis of the differentially expressed Rho family GTPases genes such as Rac3, RhoBTB1, RhoBTB3, RhoC, RhoV, RhoB, RhoBTB2, RhoF, RhoQ, and RhoU in non-M3 AML patients. The patients were grouped into gene expression high group and gene expression low group based on the median expression values of the above genes. The overall survival (OS) of patients with non-M3 AML was associated with the mRNA expression levels of RhoBTB1, RhoBTB3, RhoC, and RhoF, as indicated by the survival curve (log‑rank test, *P*<0.05) (**Figure 2A-D**). The patients with non-M3 AML with increased RhoBTB1 and RhoBTB3 mRNA expression levels or decreased RhoC and RhoF mRNA expression levels were predicted to have favorable OS. A similar prognostic impact of RhoBTB3 expression was also present in patients with non-M3 AML in another independent cohort GSE71014 (**Figure 2E**). Therefore, relatively high expression of RhoBTB3 may represent a favorable prognostic factor for non-M3 AML. Thus, we focused on RhoBTB3 gene expression in subsequent analyses and further verified that RhoBTB3 is differentially expressed in AML and contrast group using qRT-PCR (**Figure 3**).

### Associations of RhoBTB3 level with clinical and molecular features

The non-M3 AML patients from TCGA dataset were separated into chemotherapy group and allo-HSCT group based on treatment regimens. The patients in each group were further subgrouped according to median expression levels of RhoBTB3. The associations between RhoBTB3 expression levels and clinical characteristics as well as molecular features in both groups are shown in **Table 1**. In chemotherapy group, high RhoBTB3 subgroup had more favorable karyotype and more female than low RhoBTB3 subgroup. Two subgroups of patients with chemotherapy revealed different distribution characteristics of the FAB subtype. In allo-HSCT group, high RhoBTB3 subgroup had younger patients than low RhoBTB3 group. But the patient was divided into different age groups based on 60 years old, no difference was found in the subgroups with RhoBTB3 expression level.

### Prognostic value of RhoBTB3 expression in non-M3 AML

**Figure 4:**
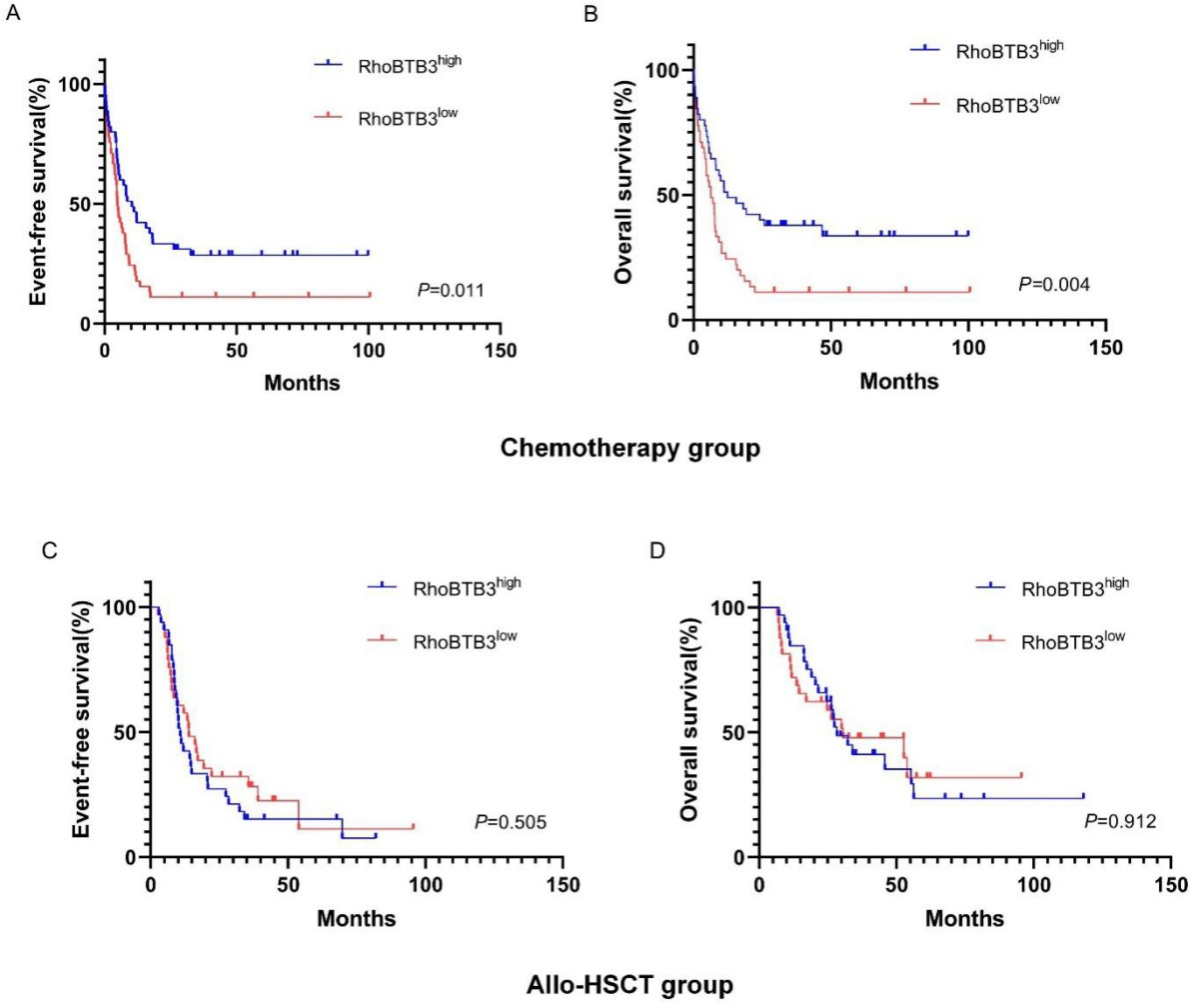
** Kaplan-Meier survival analysis of EFS and OS between chemotherapy group and allo-HSCT groups.** (**A, B**) In chemotherapy group (n=91), high RhoBTB3 subgroup had longer EFS and OS than low subgroup. (**C, D**) In allo-HSCT group (n=66), no significant difference for EFS and OS were found in high and low RhoBTB3 subgroup analysis.

In chemotherapy group, high RhoBTB3 subgroup showed better EFS and OS (both *P*<0.05) than low RhoBTB3 subgourp (**Figure 4A-4B**). However, RhoBTB3 expression were not associated with EFS and OS in allo-HCST patients (**Figure 4C-4D**). The findings indicated that high RhoBTB3 might be a beneficial factor in non-M3 AML patientis that administered chemotherapy treatment.

In chemotherapy group, results from multivariate Cox analysis revealed that WBC ≥100×10^9^/L, age ≥60 years, and mutation in TP53 were unfavorable prognostic factors for both EFS and OS (*P*<0.05, **Table 2**), while high RhoBTB3 expression was determined as an independent favorable prognositc factor for both EFS and OS (*P*<0.05, **Table 2**). In addition, ASXL1 mutation turned out an independent poor prognostic factor for OS only (*P*<0.05, **Table 2**).

In allo-HSCT group, results from multivariate Cox analysis demonstrated that FLT3-ITD was independently associated with worse EFS (*P*<0.05, **Table 3**). TP53 mutation and FLT3-ITD were independently associated with worse OS (*P*<0.05, **Table 3**).

**Figure 5:**
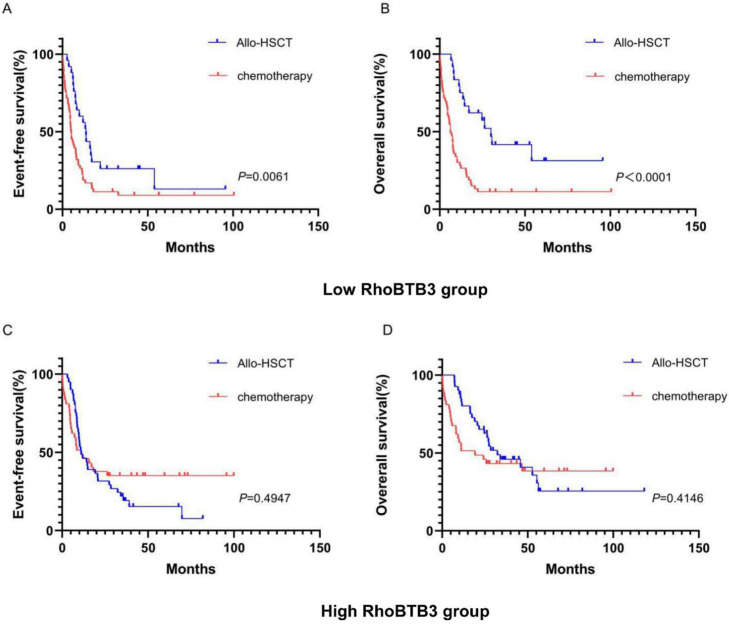
** Kaplan-Meier survival analysis of EFS and OS in low RhoBTB3 group and high RhoBTB3 group. (A, B)** In low RhoBTB3 group, patients receiving allo-HSCT (n=53) showed significantly better EFS and OS than who received chemotherapy (n=25). **(C, D)** In high RhoBTB3 group, no significant difference of EFS and OS were found between patients receiving allo-HSCT (n=41) and chemotherapy (n=38).

To explore whether the allo-HSCT treatment could overcome the poorer prognosis caused by down-regulation of RhoBTB3 expression, patients with non-M3 AML in the TCGA database were delivered into two groups (high RhoBTB3, n =79, low RhoBTB3, n =78) according to the median values of RhoBTB3 expression. In the low RhoBTB3 expression group, patients who received allo-HSCT treatment had significantly longer EFS (*P*<0.05, **Figure 5A**) and OS (*P*<0.05, **Figure 5B**) than the patients with just chemotherapy treatment only. However, we found no differences in EFS and OS between the allo-HSCT subgroup and the chemotherapy subgroup in the high RhoBTB3 expression group (**Figure 5C and Figure 5D**). We speculate allo-HSCT may overcome the adverse prognostic effects that are related to downregulated RhoBTB3 expression in AML.

### GO terms and KEGG pathway enrichment analysis of DEGs between RhoBTB3 high versus RhoBTB3 low samples

**Figure 6:**
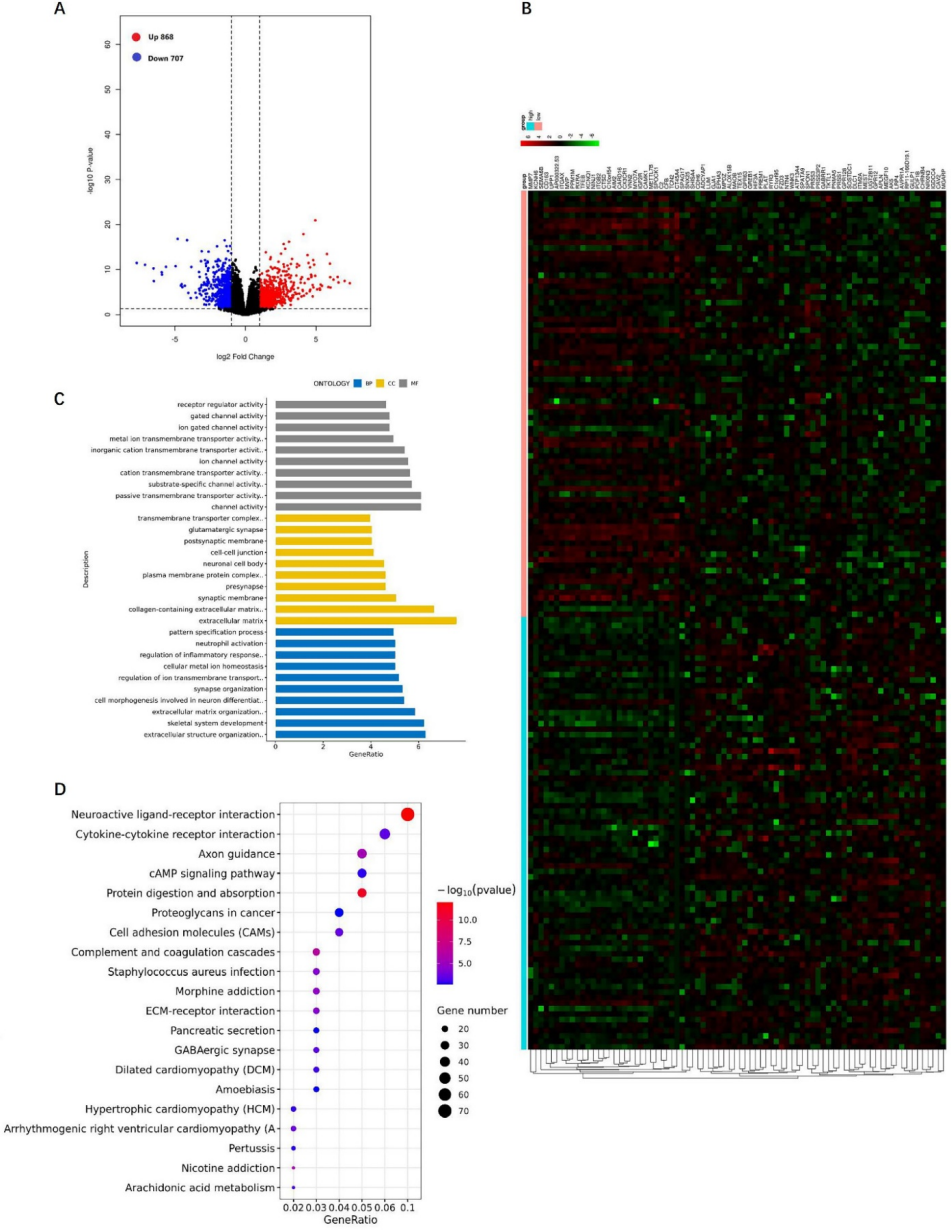
** DEGs between the RhoBTB3 high versus RhoBTB3 low groups and GO and KEGG analysis. (A)** Volcano plot of DEGs of the RhoBTB3 high group versus the RhoBTB3 low group. Red dots: up-regulated genes; Blue dots: down-regulated genes; Black dots: genes with no significant changes. **(B)** Heatmap of the top 40 up-regulated genes and the top 40 down-regulated genes. Red: high expression; Black: intermediate expression; Green: low expression. **(C)** GO analysis of DEGs. **(D)** KEGG pathway analysis of DEGs.

We analyzed the DEGs of the RhoBTB3 high group versus the RhoBTB3 low group among non-M3 AML cases in TCGA. The results revealed that the expression level of RhoBTB3 was positively associated with 868 up-regulated genes and 707 down-regulated genes (*P*< 0.05 and | log2FC |> 1, **Figure 6A**). The heatmap showed the top 40 upregulated and 40 downregulated genes (**Figure 6B**). We analyzed DEGs using enriched GO terms and KEGG pathways. In the GO analysis, most DEGs among the molecular function terms were enriched in passive transmembrane transporter activity (GO: 0022803) and channel activity (GO: 0015267) (**Figure 6C**). Among the cellular component terms in the GO analysis, most DEGs were enriched in the extracellular matrix (GO: 0031012) as well as collagen-containing extracellular matrix (GO: 0062023) (**Figure 6C**). Among the terms of biological process in the GO analysis, most DEGs were enriched in skeletal system development (GO: 0001501) and extracellular structure organization (GO: 0043062) (**Figure 6C**). In the KEGG analysis results, neuroactive ligand-receptor interaction (hsa04080) and cytokine-cytokine receptor interaction (hsa04060), as well as protein digestion and absorption (hsa04974) were the most enriched pathways (**Figure 6D**).

### PPI analysis of DEGs

We applied STRING to develop the protein-protein interaction (PPI) network by using the top 40 up-regulated genes, the top 40 down-regulated genes, and RhoBTB3. In this PPI network we have found seven genes that are directly correlated with RhoBTB3. EphA3 and SPATA9 have positive expression correlation with RhoBTB3, while MYO7A, Ninj1, IGF2R, METTL7B, and CAMK1 present negatively relationship with RhoBTB3.

## Discussion

Rho family GTPases members play roles in cell proliferation and motility, cytoskeletal regulation, cell polarity establishment, and transcriptional regulation 3-5. Some studies have found that this gene family is also important in tumorigenesis and development 22. They can be involved in promoting tumors as well as suppressing tumors 23. So far, the associations among overall survival, clinical characteristics, and Rho family GTPases expression in AML remain unknown. Our study showed that compared with normal bone marrow samples, the mRNA expression of RhoB, RhoBTB2, RhoF, RhoQ, and RhoU was significantly increased in AML bone marrow samples, while the mRNA expression levels of Rac3, RhoBTB1, RhoBTB3, RhoC, and RhoV was decreased in AML bone marrow. According to current studies, RhoC promotes the process of progression of some cancers, including ovarian cancer as well as head and neck cancer 24, 25. Rac3-KO mice presented higher survival rates in CML and ALL, suggesting a potential oncogenic role in cancer 26, 27. RhoB is often downregulated in malignancies like lung cancer and gastric cancer by suppressing the process of proliferation, migration, as well as invasion of tumor cells 28-30. RhoBTB1 and RhoBTB2 are reduced in some tumors and known as tumor suppressors for their involvement in the cell cycle and apoptosis 31, 32. The expression level of RhoBTB3 is significantly lower in tumor tissues of breast, kidney, lung, uterus and ovary than that in normal tissues according to a cancer profiling array 33. Further experiments will be needed to verify the particular functional significance of these family genes in AML.

Among above genes with significantly different expression between AML bone marrow and healthy bone marrow, we found that RhoBTB3 was associated with OS in TCGA non-M3 AML patients by survival curve analysis. The prognostic value of RhoBTB3 was verified in another dataset GSE71014. Then we verified the differential expression of RhoBTB3 on AML bone marrow and nonleukemia/non-tumor bone marrow by RT-qPCR. Similar to our findings, human renal carcinomas express a low level of RhoBTB3, and RhoBTB3 deficiency can significantly enhance the Warburg effect as well as accelerate xenograft growth 34. RhoBTB3 is also a Golgi-associated protein that is critical in keeping structure of Golgi and homeostasis of cells. The deficiency of RhoBTB3 could result in accumulation of cellular substrates which is important for cell degradation, causing an instability of cellular homeostasis, thus ultimately leading to cancers 35. The correlation between RhoBTB3 and cell cycle regulation has been reported recently 36. The mechanism that RhoBTB3 could suppress the development of cancer might be associated its role as the adaptor protein of Cullin3-dependent RING-E3 ubiquitin ligase complex 36. Cullin3 is a kind of scaffolding protein that specifically recognize and mediate ubiquitination of the substrate through combination with adaptor protein with a BTB domain-bearing protein 37. Studies by Lu et al. have revealed that RhoBTB3 could target Cyclin E and mediate its ubiquitination via Cullin3-dependent RING-E3 ubiquitin ligase complex, thus preventing cells from transition from phase S to G2 phase. Downregulation of RhoBTB3 could lead to fragmentation Golgi fragmentation and increased levels of Cyclin E which can regulates cell cycle 36. Previous studies have found increased expression level of Cyclin E in AML, which promote the development of AML 38. Studies by Huang et al showed that Notopterol could induce G0/G1 arrest as well as apoptosis of human AML HL-60 cells through regulation of CDK2 and Cyclin E expression 39. Above all, decreased expression level of RhoBTB3 in AML could result in reduced uquibitination of Cyclin E, enhanced expression of Cyclin E as well as progression of the development of AML. In Cox analysis, we found that higher expression of RhoBTB3 was independently associated with favorable prognosis of AML patients who received chemotherapy, and the allo-HSCT treatment may overcome unfavorable prognosis of AML patients with low RhoBTB3 expression. The subgroup of low RhoBTB3 expression that receiving allo-HSCT therapy had significantly longer EFS and OS than chemotherapy subgroup. However, in patients with high RhoBTB3 expression no superiorities of the allo-HSCT group was found compared with chemotherapy. The above results remind us that AML patients with low RhoBTB3 expression may be strongly recommended for early allo-HSCT. However, allo-HSCT may be limited in improving survival in AML patients with high expression of RhoBTB3. Studying the relationship between RhoBTB3 and the pathogenesis and prognosis of AML will help us choose more appropriate treatment options for heterogeneous AML patients. Additionally, the prognosis of patients with TP53 mutations is poor whether they undergo chemotherapy or allo-HSCT, which is in line with previous studies, people try to use demethylation therapy and immunomodulatory therapeutic to improve the prognosis of this type of AML patients 40.

We further analyzed DEGs related to RhoBTB3. We found that 868 up-regulated genes and 707 down-regulated genes were closely related with the expression of RhoBTB3. GO analysis showed that passive transmembrane transporter activity, channel activity, extracellular matrix collagen-containing extracellular matrix, skeletal system development, and extracellular structure organization were markedly enriched among DEGs associated with RhoBTB3 expression. KEGG pathways were mainly enriched in neuroactive ligand-receptor interactions, cytokine-cytokine receptor interactions, protein digestion and absorption, axon guidance, and cAMP signaling pathways. The heatmap showed the top 40 up-regulated genes as well as top 40 down-regulated genes that were positively associated with high RhoBTB3 expression. Among them, we have found 7 genes that are directly correlated with RhoBTB3 expression in PPI network. EphA3 and SPATA9 have positive expression correlation with RhoBTB3, and MYO7A, Ninj1, IGF2R, METTL7B, and CAMK1 present negatively relationship with RhoBTB3. EphA3 and SPATA9 were lower in some tumors tissue and served as tumor‑suppressors 41. Many studies have confirmed that MYO7A, Ninj1, IGF2R, METTL7B, and CAMK1 increased expression is related to tumors 42-45. However, the correlation of these genes with RhoBTB3 has not been verified in molecular biology experiment. We need more in-depth exploration in the future.

## Conclusions

Our study analyzed the expression patterns and prognostic significance of Rho family GTPases genes in AML. RhoBTB3 is significantly downregulated in AML bone marrow compared to healthy controls. The expression of RhoBTB3 level may help us identify heterogeneous AML patients with different prognosis and choose treatment options suitable for AML patients such as chemotherapy or allo-HSCT. The function of RhoBTB3 in AML is worthy of further study.

## Figures and Tables

**Figure 7:**
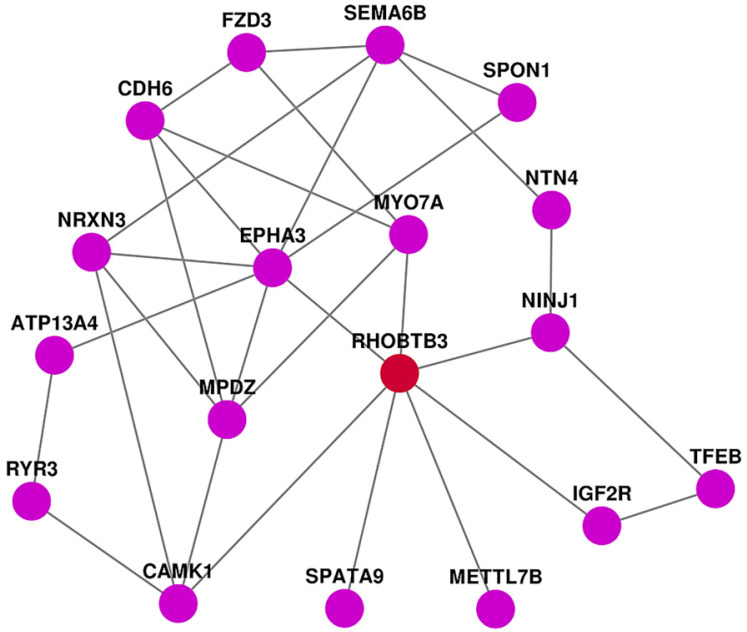
PPI network analysis.

**Table 1:** Comparison of clinicopathological parameters with RhoBTB3 expression in patients (n=157)

Variables	Chemotherapy group			Allo-HSCT group		
	High RhoBTB3 (*n*=46)	Low RhoBTB3 (*n*=45)	*P*	High RhoBTB3 (*n*=33)	Low RhoBTB3 (*n*=33)	*P*
Age/years, median (range)	65 (22-76)	66 (25-88)	0.406	49 (18-65)	55 (21-72)	0.030
**Age group/n (%)**			0.717			0.097
<60 years	17 (36.96)	15 (33.33)		27 (81.82)	21 (63.64)	
≥60 years	29 (63.04)	30 (66.67)		6 (18.18)	12 (36.36)	
**Gender/n (%)**			0.046			0.319
Female	27 (58.70)	17 (37.78)		12 (36.36)	16 (48.48)	
Male	19 (41.30)	28 (62.22)		21 (63.64)	17 (51.52)	
WBC (×10^9^/L)/median (range)	13.7 (1.7-297.4)	16 (0.7-137.2)	0.738	32.4 (0.6-223.8)	29.7 (0.8-118.8)	0.423
BM blast /(%), median (range)	69.5 (30-99)	75 (32-98)	0.433	70 (30-100)	72 (39-97)	0.677
PB blast/(%), median (range)	47 (0-98)	13 (0-87)	0.012	62 (0-96)	45 (0-87)	0.076
**FAB subtype/n (%)**			0.043			0.109
M0	1 (2.17)	6 (13.33)		2 (6.06)	7 (21.21)	
M1	15 (32.61)	6 (13.33)		13 (39.39)	10 (30.30)	
M2	14 (30.43)	8 (17.78)		11 (33.33)	5 (15.15)	
M4	10 (21.74)	12 (26.67)		5 (15.15)	7 (21.21)	
M5	4 (8.70)	11 (24.44)		0 (0.00)	3 (9.09)	
M6	0 (0.00)	1 (2.22)		1 (3.03)	0 (0.00)	
M7	1 (2.17)	1 (2.22)		0 (0.00)	1 (3.03)	
NC	1 (2.17)	0 (0.00)		1 (3.03)	0 (0.00)	
**RUNX1/n (%)**			0.440			0.720
Widtype	43 (93.48)	40 (88.89)		29 (87.88)	28 (84.85)	
Mutation	3 (6.52)	5 (11.11)		4 (12.12)	5 (15.15)	
**TP53/n (%)**			0.316			0.555
Widtype	42 (91.30)	38 (84.44)		32 (96.97)	31 (93.94)	
Mutation	4 (8.70)	7 (15.56)		1 (3.03)	2 (6.06)	
**ASXL1/n (%)**			0.570			1.000
Widtype	44 (95.65)	44 (97.78)		32 (96.97)	32 (96.97)	
Mutation	2 (4.35)	1 (2.22)		1 (3.03)	1 (3.03)	
**NPM1/n (%)**			0.100			0.057
Widtype	35 (76.09)	27 (60.00)		27 (81.82)	20 (60.61)	
Mutation	11 (23.91)	18 (40.00)		6 (18.18)	13 (39.39)	
**FLT3-ITD/n (%)**			0.146			0.085
Absence	37 (80.43)	41 (91.11)		28 (84.85)	22 (66.67)	
Presence	9 (19.57)	4 (8.89)		5 (15.15)	11 (33.33)	
**biCEBPA/n (%)**			0.157			0.076
Absence	44 (95.65)	45 (100.00)		30 (90.91)	33 (100.00)	
Presence	2 (4.35)	0 (0.00)		3 (9.09)	0 (0.00)	
**Karyotype/n (%)**			0.007			0.244
Favorable	8 (17.39)	0 (0.00)		4 (12.12)	1 (3.03)	
Intermediate	31 (67.39)	32 (71.11)		23 (69.70)	22 (66.67)	
Adverse	7 (15.22)	13 (28.89)		6 (18.18)	10 (30.30)	

**Abbreviations**: WBC: white blood cell; PB: peripheral blood; BM: bone marrow; FAB: French American British.

**Table 2:** Univariate and Multivariate analyses in patients (n=91) with chemotherapy

	EFS		OS	
	HR (95%CI)	*P*-value	HR (95%CI)	*P*-value
**Univariate analysis**				
RhoBTB3 (High vs Low)	0.569 (0.356-0.909)	0.018	0.518 (0.320-0.838)	0.007
age (≥60 vs <60)	3.375 (1.937-5.879)	<0.001	3.156 (1.792-5.560)	<0.001
Gender (Male vs Female)	1.036 (0.655-1.640)	0.879	1.168 (0.730-1.868)	0.518
WBC (≥100 vs <100×10^9^)	1.247 (0.620-2.508)	0.537	1.328 (0.658-2.681)	0.428
RUNX1 mutation(Yes vs No)	1.539 (0.736-3.221)	0.252	1.673 (0.798-3.508)	0.173
TP53 mutation (Yes vs No)	3.217 (1.645-6.292)	0.001	3.136 (1.608-6.115)	0.001
ASXL1 mutation (Yes vs No)	1.840 (0.573-5.912)	0.306	1.857 (0.579-5.956)	0.298
NPM1 mutation (Yes vs No)	1.211 (0.742-1.975)	0.443	1.046 (0.631-1.733)	0.861
FLT3-ITD mutation (Yes vs No)	1.057 (0.556-2.008)	0.866	0.832 (0.413-1.676)	0.606
biCEBPA mutation (Yes vs No)	0.388 (0.054-2.798)	0.348	0.427 (0.059-3.080)	0.399
**Multivariate analysis**				
RhoBTB3 (High vs Low)	0.576 (0.359-0.922)	0.022	0.501 (0.307-0.818)	0.006
age (≥60 vs <60)	3.607 (1.973-6.595)	<0.001	3.352 (1.817-6.187)	<0.001
WBC (≥100 vs <100×10^9^)	2.361 (1.112-5.011)	0.025	2.495 (1.171-5.320)	0.018
TP53 mutation (Yes vs No)	2.341 (1.173-4.670)	0.016	2.503 (1.247-5.026)	0.010
ASXL1 mutation (Yes vs No)	-	-	3.343 (1.004-11.134)	0.049

**Table 3:** Univariate and Multivariate analyses in patients (n=66) with Allo-HSCT

	EFS		OS	
	HR (95%CI)	*P*-value	HR (95%CI)	*P*-value
Univariate analysis				
RhoBTB3 (High vs Low)	1.200 (0.701-2.055)	0.506	1.036 (0.552-1.946)	0.912
age (≥60 vs <60)	0.845 (0.459-1.556)	0.588	1.195 (0.579-2.468)	0.629
Gender (Male vs Female)	0.954 (0.550-1.657)	0.869	0.805 (0.427-1.516)	0.502
WBC (≥100 vs <100×10^9^)	1.537 (0.602-3.924)	0.369	2.212 (0.756-6.470)	0.147
RUNX1 mutation (Yes vs No)	0.762 (0.325-1.787)	0.532	1.256 (0.485-3.249)	0.639
TP53 mutation (Yes vs No)	1.650 (0.509-5.346)	0.404	4.559 (1.319-15.755)	0.016
ASXL1 mutation (Yes vs No)	0.679 (0.164-2.803)	0.593	0.518 (0.071-3.789)	0.517
NPM1 mutation (Yes vs No)	0.863 (0.475-1.567)	0.628	0.904 (0.450-1.816)	0.776
FLT3-ITD mutation (Yes vs No)	1.934 (1.041-3.591)	0.037	2.029 (0.969-4.247)	0.061
biCEBPA mutation (Yes vs No)	0.617 (0.150-2.546)	0.504	0.712 (0.169-2.989)	0.642
Multivariate analysis				
TP53 mutation (Yes vs No)	-	-	5.732 (1.611-20.398)	0.007
FLT3-ITD mutation (Yes vs No)	1.934 (1.041-3.591)	0.037	2.288 (1.076-4.866)	0.031
